# Quick Systemic Lupus Activity Questionnaire (Q-SLAQ): a simplified version of SLAQ for patient-reported disease activity

**DOI:** 10.1136/lupus-2020-000471

**Published:** 2021-05-09

**Authors:** Elisabet Svenungsson, Iva Gunnarsson, Vera Illescas-Bäckelin, Estelle Trysberg, Andreas Jönsen, Dag Leonard, Christopher Sjöwall, Susanne Pettersson

**Affiliations:** 1 Division of Rheumatology, Department of Medicine Solna, Karolinska Institutet, Stockholm, Sweden; 2 Rheumatology, Karolinska University Hospital, Stockholm, Sweden; 3 Department of Rheumatology and Inflammation Research, University of Gothenburg, Goteborg, Sweden; 4 Department of Clinical Sciences Lund, Rheumatology, Lund University, Lund, Sweden; 5 Department of Medical Sciences, Science for Life Laboratory, Rheumatology, Uppsala University, Uppsala, Sweden; 6 Department of Biomedical and Clinical Sciences, Division of Inflammation and Infection/Rheumatology, Linköping University, Linköping, Sweden; 7 Department of Neurobiology, Care Sciences and Society, Division of Physiotherapy, Karolinska Institutet, Stockholm, Sweden

**Keywords:** lupus erythematosus, systemic, outcome assessment, health care, autoimmune diseases

## Abstract

**Objectives:**

Most indices of disease activity in SLE combine physicians’ assessments and laboratory tests. However, there is also a need to capture patients’ perspectives of disease activity. Consequently, we need new, preferably quick and easy instruments to collect this information, which can be very useful for online consultations and registry purposes. We compared patients’ assessments of SLE disease impact/activity, as reported by a shorter version of the Quick Systemic Lupus Activity Questionnaire (Q-SLAQ), with physicians’ assessments using SLE Activity Measure (SLAM) and SLE Disease Activity Index (SLEDAI-2K) and with the original Systemic Lupus Activity Questionnaire (SLAQ).

**Methods:**

Patients with SLE (n=115), with a disease duration of 15 years (IQR 17), completed the Q-SLAQ prior to physicians’ assessments by SLAM and SLEDAI-2K. A second set of patients (n=85) with similar characteristics filled out Q-SLAQ and SLAQ. Spearman’s ρ correlations were explored between patients’ total Q-SLAQ and subscales (Symptom Score, Patient’s Global Disease Activity) and physicians’ SLAM and SLEDAI-2K, with and without laboratory items (SLAM-nolab and SLEDAI-2K-nolab) and SLAQ. Corresponding items in Q-SLAQ and SLAM were compared.

**Results:**

Correlations between patients’ and physicians’ assessments were higher for SLAM-nolab (total Q-SLAQ, ρ=0.71; Symptom Score, ρ=0.67; and Patient’s Global Disease Activity, ρ=0.68) than for the original SLAM (total Q-SLAQ, ρ=0.53; Symptom Score, ρ=0.50; and Patient’s Global Disease Activity, ρ=0.53). Regarding specific symptoms, fatigue (ρ=0.72) and alopecia (ρ=0.71) correlated best, while pulmonary/respiratory symptoms correlated least (ρ=0.19, p=0.039). Physicians assessment with SLEDAI-2K-nolab correlated weakly with patients’ assessments (total Q-SLAQ, ρ=0.30; Symptom Score, ρ=0.30; and Patient’s Global Disease Activity, ρ=0.36). Bivariate correlations between Q-SLAQ and SLAQ were good (ρ=0.82–0.96).

**Conclusions:**

Q-SLAQ and the original SLAQ performed equally well, demonstrating that the shorter Q-SLAQ can safely be used to monitor patients’ perception of disease impact/activity. We also noted an intriguing discrepancy between physicians’ and patients’ evaluations of pulmonary/respiratory symptoms, which requires further investigations.

**Key messages**

**What is already known about this subject?**

- The Systemic Lupus Activity Questionnaire (SLAQ) is constructed to be used in epidemiological studies, but it is extensive, and some questions are difficult for patients to answer.

**What does this study add?**

- The shorter version of the Quick Systemic Lupus Activity Questionnaire (Q-SLAQ) has less items than SLAQ but similar correlations between patients and physician’s assessment of disease activity.
- A profound discrepancy regarding patients’ assessment versus doctors’ assessment of thoracic and respiratory symptoms is demonstrated.

**How might this impact on clinical practice or future developments?**

- The Q-SLAQ can safely be used to monitor patients’ perception of disease and is thereby well suited for frequent use in clinical practice and registries.
- The discrepancy between physicians’ and patients’ evaluation of pulmonary/respiratory manifestations is a finding that needs clinical attention.

## Introduction

SLE is a chronic inflammatory disease with multiple manifestations. The disease has a variable course where periods of flare-ups and remissions intervene, though some patients also have a more persistently active disease.^1^ High disease activity, but also side effects of treatments, contributes to significant organ damage over time.^2^ With the over-reaching goal to prevent organ damage, standardised indices based on more or less time-consuming questionnaires filled out by doctors are used to monitor disease activity and to evaluate treatment in general practice and in clinical trials. However, there is presently no consensus on which questionnaires best reflect disease activity, and knowledge is limited regarding the role of patient-reported outcome measures (PROMs) in the assessment of SLE disease activity.^3^ Continuous research to improve standardised tools to monitor disease activity and treatment outcomes is therefore of major importance in SLE.^4^

The Systemic Lupus Activity Questionnaire (SLAQ) is an extensively translated tool that captures patients’ assessments of SLE-related symptoms and disease activity, with good correlations to physicians’ assessments of SLE disease activity.^5–7^ The SLAQ is constructed to be used in epidemiological studies and aims to capture the multitude of potential organ manifestations of SLE over the last 3 months, resulting in an extensive questionnaire with 26 items for patients to consider and respond to. However, in a recent study, we found that some of the questions in the SLAQ were difficult to answer and had poor correlation to physicians’ assessments of symptom activity.^7^ Interestingly, this was most evident for patients with short disease duration. In clinical practice, which nowadays incorporates a growing share of online consultations, it is important to have an ‘easy to fill in’ tool which captures PROMs adequately for the majority of patients. However, to be useful in repeated consultations during disease flares, the instrument should cover a shorter time period than the original SLAQ, which covers the previous 3 months. Such an instrument would also be very valuable for clinical registry purposes. Based on our previous experience, we therefore revised the Swedish version of the SLAQ into a shorter version, which is easy for the patients to answer (Quick Systemic Lupus Activity Questionnaire (Q-SLAQ).

In the present study, we evaluate the performance of Q-SLAQ in comparison to SLE Activity Measure (SLAM),^8^ SLE Disease Activity Index (SLEDAI-2K)^9 10^ and the original SLAQ.^5 7^

## Patients and methods

### Patients

Patients with SLE were consecutively included at clinical visits. Since healthcare, including the use of questionnaires, might vary between regional centres, we recruited experienced physicians and patients from five tertial referral rheumatology specialist centres in Sweden. All participants fulfilled at least four of the American College of Rheumatology criteria for SLE.^11^ The patients completed the Q-SLAQ prior to visiting the rheumatology specialist centre for a medical examination. Rheumatologists, who were blinded to the patients’ Q-SLAQ results, filled out SLAM^8^ and SLEDAI-2K^9^ during the clinical consultation. Additionally, a different set of patients completed both the Q-SLAQ and the Swedish version of the original SLAQ,^7^ with or without physicians’ assessments of SLAM according to clinical practice. The patients in the additional data collection were comparable to the first data group, according to age and disease duration.

### Questionnaires

The original version of the SLAQ includes 26 items that capture patients’ assessments of SLE-related symptoms and disease activity. The questionnaire has four possible scoring systems: (1) the total SLAQ score grades the severity of 24 symptoms on a scale of 0–47; (2) the Symptom Score is the sum of the non-graded presence (1) or absence (0) of symptoms and is rated on a scale of 0–24; (3) the severity of lupus flare-ups is rated on a scale of 0–3; and (4) the Patients’ Global Disease Activity is rated on a numerical rating scale of 0–10. The translation process from the original SLAQ to the Swedish version of the SLAQ is previously described by Pettersson *et al*.^7^ This version was used for comparison in the additional set of patients. The previous study among Swedish patients showed excellent to good internal consistency (Cronbach’s alpha) of the Symptom Score (0.907) and the SLAQ score (0.862).^7^ However, the question of flare-ups seemed to be the most difficult for patients to answer, and additionally, the analyses of Cronbach’s alpha suggested that the removal of the epilepsy symptom item slightly improved the scale. Moreover, the only symptom item with no correlation between the patients’ and the physicians’ assessments, in our previous study, was neurological/stroke syndrome. Based on these results, these two symptom items (epilepsy and neurological/stroke syndrome) were removed as was the question about flare-ups. After discussions with patients’ representative research partners, we added one symptom item, dryness in the mouth and eyes (sicca symptoms). Furthermore, a discussion of clinical relevance was conducted with an executive group of senior rheumatologists with extensive experience working with SLE, and the reduced version of the SLAQ was determined as clinically relevant. Finally, the time frame of the patients’ assessment was decreased from the original 3 to 1 month to be more comparable to the physicians’ assessment of SLAM, to be more useful for clinical consultations during flares and also to improve recall accuracy.

This procedure resulted in a new, revised and tentative version of the Swedish questionnaire, the Q-SLAQ, covering patients’ report of 19 symptom items and one global activity item. Congruent with previous studies, excluding the question of flare-ups, the following three scorings were analysed in the present study. First, the calculation of the Total Q-SLAQ Score was based on the algorithm that was previously developed by Karlson *et al*.^5 7^ This algorithm converts distress from joints, muscles, lungs and cognitive impairment, each of which were represented by two questions, while three questions deal with skin/mucosal distress combined into one item, respectively. This results in a questionnaire that captures distress from 13 areas (weight loss, fatigue, fever, lymphadenopathy, dryness of the eyes/mouth, myalgia/myositis, arthralgia/arthritis, skin/mucosal, alopecia, pulmonary, abdominal pain, headaches and cognitive dysfunction). Second, the simple calculation of the Q-SLAQ Symptom Score was used, which is the sum of the non-graded presence regardless of mild, moderate or severe (1) or absence (0) of symptoms among the 19 investigated symptom items. Third, the Patient’s Global Disease Activity was assessed with a single item.

To summarise, the Total Q-SLAQ Score is a summary score on a scale of 0–37; the Symptom Score is a summary score on a scale of 0–19 and the Patient’s Global Disease Activity is scored on a scale of 0–10 (online supplemental). High values indicate greater perceived disease impact/distress.

10.1136/lupus-2020-000471.supp1 Supplementary data

10.1136/lupus-2020-000471.supp2 Supplementary data

### Disease activity evaluated by the physicians

In both questionnaires, SLAM and SLEDAI-2K, used by the physicians, high values indicate more disease activity. SLAM covers and grades clinical symptoms and laboratory variables, in nine organ systems during the previous month (score range of 0–83).^8^ A SLAM score of >6 is considered clinically important and is used as an indication to start medical treatment.^12^ The SLEDAI-2K includes presence versus absence of symptoms in nine organ systems represented by 16 clinical and 8 laboratory items (score range 0–105).^10^ Since the patient’s self-assessment of Q-SLAQ does not include any laboratory variables, we calculated and evaluated SLAM and SLEDAI-2K, with and without laboratory items (SLAM-nolab, score range 0–61; SLEDAI-2K-nolab, score range 0–83).

### Statistics

For statistical calculations, the Statistical Package for the Social Sciences (SPSS, Chicago, IL, USA), version 20 was used. The scales used for the evaluations in this study were primarily ordinal; thus, median, IQR and non-parametric tests were used. A *p*-value of <0.05 was considered statistically significant. The internal consistency of the Symptom Score (19 items) and the Total Q-SLAQ Score (13 items) was calculated using Cronbach’s alpha.^13^ To explore the criterion validity, the relationship between the SLAQ and previously established measurements was assessed.^13^ Bivariate correlations with Spearman’s correlations were used to compare the patients’ assessments (Total Q-SLAQ Score, Symptom Score and Patient’s Global Disease Activity) and physicians’ assessments (SLAM, SLAM-nolab, SLEDAI-2K and SLEDAI-2K-nolab) of disease activity; additionally, the individual symptom items from the SLAQ were compared with the corresponding items on SLAM. In the second data collection bivariate correlations with Spearman’s correlations were used to explore the subscales of patients’ assessments of both Q-SLAQ and the Swedish version of SLAQ (Total SLAQ Score, Symptom Score and Patient’s Global Disease Activity). Bivariate correlations for both these questionnaires were also analysed with using only the physicians’ assessments (SLAM-nolab) and not including the laboratory evaluations. The correlation coefficients were evaluated using Colton’ guidelines.^14^ The Mann-Whitney U test was used for between-group comparisons.

An overview of the results from Q-SLAQ and previous studies of the original version of SLAQ^5 6^ as well as the Swedish version of SLAQ^7^ is presented in the online supplemental material.

## Results

This study analysed 115 paired assessments conducted by both patients and physicians. The patient’s characteristics (87% women) are presented in table 1. A majority (70%) of the patients had low disease activity as captured by SLAM (≤6). The response rate was ≥95% for all the individual items. Cronbach’s alpha results were 0.905 for the Symptom Score (19 items) and 0.893 for the Total Q-SLAQ Score (13 items). For comparisons, performances of Q-SLAQ, Swe-SLAQ^7^ used in a study with similar cultural context and the original SLAQ^5^ are presented in the online supplemental data.

**Table 1:** Characteristics of the 115 participants with SLE (women 87%)

	Median	IQR	Min–Max*
Age (years)	43	32–56	18–77
Disease duration†	15	7–24	0–49
Patients’ assessment			
Total Q-SLAQ‡	10	5–17	0–35
Symptom Score‡	8	4–13	0–19
Patient’s global disease activity§	4	1–7	0–9
Physician’s assessment			
SLAM-nolab	3	1–6	0–14
SLAM	4	2–9	0–19
SLEDAI-2K-nolab	0	0–2	0–16
SLEDAI-2K	2	2–5	0–21

*Min–max indicates range from lowest to highest.

†Missing, n=33.

‡Without the sicca item.

§Missing, n=5.

Q-SLAQ, Quick Systemic Lupus Activity Questionnaire; SLAM, SLE Activity Measure; SLAM-nolab, Systemic Lupus Activity Measure without laboratory parameters; SLEDAI-2K, SLE Disease Activity Index; SLEDAI-2K-nolab, SLE Disease Activity Index without laboratory parameters.

### Associations between patients’ and physicians’ assessments

Bivariate correlation between the patients’ and physicians’ assessments is described in table 2. The summary scores (the Total Q-SLAQ Score and the Symptom Score) were explored with and without the new item sicca symptom. Overall, the strongest correlations were observed between the physicians’ assessment using SLAM-nolab and all the scoring results for the Q-SLAQ without the sicca symptom. The strongest correlation was observed between the physicians’ SLAM-nolab and the patients’ Total Q-SLAQ scores (ρ=0.71, p<0.001). No significant correlations were identified between the patients’ and physicians’ assessments when using SLEDAI-2K (ρ<0.09 for all).

**Table 2:** Correlations* between patients’ self-assessment of SLE disease activity and physician’s assessment (n=115)

	Coefficient*	P value
SLAM-nolab versus		
Total Q-SLAQ	0.709	<0.001
Total Q-SLAQ with sicca	0.690	<0.001
Symptom Score	0.680	<0.001
Symptom Score with sicca	0.674	<0.001
Patient’s global disease activity	0.683	<0.001
SLAM versus		
Total Q-SLAQ	0.528	<0.001
Total Q-SLAQ with sicca	0.505	<0.001
Symptom Score	0.496	<0.001
Symptom Score with sicca	0.488	<0.001
Patient’s global disease activity	0.529	<0.001
SLEDAI-2K nolab versus		
Total Q-SLAQ	0.303	0.001
Total Q-SLAQ with sicca	0.274	0.004
Symptom Score	0.301	0.001
Symptom Score with sicca	0.292	0.002
Patient’s global disease activity	0.361	<0.001
SLEDAI-2K versus		
Total Q-SLAQ	−0.046	0.622
Total Q-SLAQ with sicca	−0.084	0.371
Symptom Score	−0.028	0.766
Symptom Score with sicca	−0.042	0.656
Patient’s global disease activity	0.086	0.374

*Spearman correlation.

Q-SLAQ, Quick Systemic Lupus Activity Questionnaire; SLAM, SLE Activity Measure; SLAM-nolab, Systemic Lupus Activity Measure without laboratory parameters; SLEDAI-2K, SLE Disease Activity Index; SLEDAI-2K-nolab, SLE Disease Activity Index without laboratory parameters.

### Patient-reported symptoms versus disease activity measures

When stratifying the participants into low disease activity (SLAM score≤6, n=80) and high disease activity (SLAM score≥7, n=35), all the Q-SLAQ subscales confirmed a distinct and significant difference (p>0.001) between the two patient groups. Participants with low disease activity had a Total Q-SLAQ median of 7 (IQR 3–13) (p<0.001) in comparison to those with high disease activity in whom the median was 15 (IQR 11–21). The Symptom Score median was 7 (IQR 3–11) vs 12 (IQR 9–15), and the Patient’s Global Disease Activity median was 2 (IQR 1–5) vs median 7 (IQR 4–8), respectively, for the high and the low disease activity patient groups. Comparing correlations in the two disease groups, respectively, we found that the correlations between patients’ and physician’s assessments were consequently higher for SLAM in the low disease activity group, but for SLEDAI-2K, they were better in the high disease activity group (table 3). We also explored proportional contribution of the laboratory parameters to the total score for all 115 patients. For SLAM, laboratory measures constituted 28% (27% in high and 29% in low disease activity group). For SLEDAI-2K, ‘the laboratory fraction’ was responsible for a greater share, 63% of the total score for all (43% in the low and 82% in the high disease activity group). We explored the individual contributions from the eight laboratory items in SLEDAI-2K, but only leucopenia was reported for a significantly higher proportion of patients with high disease activity (14%) compared with the low disease activity (4%, p=0.038, χ^2^).

**Table 3:** Spearman’s correlations between patients’ Q-SLAQ and physicians’ assessments of disease activity, stratified by low SLAM scores (≤6, n=80) vs high SLAM scores (>6, n=35)

		SLAM-nolab (SLAM score≤6)	SLAM-nolab (SLAM score>6)	SLEDAI-2K-nolab (SLAM score≤6)	SLEDAI-2K-nolab (SLAM score>6)
Total Q-SLAQ	Correlation	0.637**	0.424*	−0.099	0.298
	Sig. (two-tailed)	0.000	0.011	0.386	0.097
Total Q-SLAQ with sicca	Correlation	0.639**	0.409*	−0.081	0.285
	Sig. (two-tailed)	0.000	0.015	0.477	0.114
Symptom Score	Correlation	0.668**	0.446**	−0.037	0.463**
	Sig. (two-tailed)	0.000	0.007	0.748	0.008
Symptom Score with sicca	Correlation	0.663**	0.431**	−0.028	0.456**
	Sig. (two-tailed)	0.000	0.010	0.805	0.009
Patient’s global global disease activity	Correlation	0.622**	0.396*	0.073	0.187
	Sig. (two-tailed)	0.000	0.022	0.529	0.306

*P ≤0.05, **P ≤0.01, ***P ≤0.001.

Q-SLAQ, Quick Systemic Lupus Activity Questionnaire; Sig, significance P; SLAM, SLE Activity Measure; SLAM-nolab, Systemic Lupus Activity Measure without laboratoryparameters; SLEDAI-2K, SLE Disease Activity Index; SLEDAI-2K-nolab, SLE Disease Activity Index without laboratory parameters.

### Correlation between symptom items

All symptom items were more frequently reported by the patients than the physicians (table 4). The symptoms that most of the patients assessed as being present were fatigue (83%), arthritis/arthralgia (70%) and musculoskeletal symptoms (67%). When exploring the correlations of single items between the patients’ and physicians’ assessments, the strongest correlations were found for fatigue (ρ=0.72, p<0.001) and alopecia (ρ=0.71, p<0.001). Notably, symptoms of dyspnoea/pleuritic chest pain had the lowest correlation between patients’ and the physicians’ assessments (ρ=0.194, p=0.039). Exploring the association between the SLAM question of pleuritis and one or two of the patients’ SLAQ responses reflecting dyspnoea and/or chest pain did not improve the concordance between physicians and patients regarding pulmonary/respiratory distress.

**Table 4:** Frequency of positive responses per organ/item on patients’* and physicians’† assessments and correlations‡ between items from Q-SLAQ and SLAM-nolab (n=115)

	Positive response (%) Q-SLAQ*	Positive response (%) SLAM†	Positive response (%) SLEDAI-2K†	Correlation‡ Q-SLAQ versus SLAM	P value
Weight loss	20.0	8.7	n.i.	0.526	<0.001
Fatigue	82.6	67.0	n.i.	0.718	<0.001
Fever	21.7	7.0	4.0	0.397	<0.001
Lymphadenopathy	18.3	6.1	n.i.	0.280	0.002
Dryness eyes/mouth	55.7	n.i.	n.i.	–	–
Myalgia/myositis§	67.0	28.7	0.9	0.429	<0.001
Arthralgia/arthritis§	70.4	41.7	6.1	0.449	<0.001
Skin¶	61.7	19.1	15.9	0.231	0.014
Oral ulcer**††	33.9	8.7	5.3	0.287	0.002
Alopecia	36.5	26.1	13.9	0.714	<0.001
Pleuritic chest pain	32.5	4.3	2.6	0.194	0.039
Abdominal pain	35.7	7.8	n.i	0.463	<0.001
Headaches	51.3	27.4	0.9	0.462	<0.001
Cognitive dysfunction§‡‡	66.1	17.4	1.8	0.365	<0.001

*Q-SLAQ assessed by patients.

†SLAM and SLEDAI-2K assessed by physicians.

‡Spearman correlation.

§Cognitive dysfunction, myalgia/myositis, arthralgia/arthritis: all consists of two items on revised SLAQ but single item on SLAM and SLEDAI-2K.

¶Skin=oral ulcers, malar rash, photosensitivity (three items on Q-SLAQ, single item on SLAM and SLEDAI-2K).

**Oral ulcer as single item in both Q-SLAQ and SLEDAI-2K.

††Oral ulcer included in the question skin in the questionnaire SLAM.

‡‡Organic brain syndrome on SLEDAI-2K.

n.i., not included; Q-SLAQ, Quick Systemic Lupus Activity Questionnaire; SLAM, SLE Activity Measure; SLAM-nolab, Systemic Lupus Activity Measure without laboratory parameters; SLAQ, Systemic Lupus Activity Questionnaire; SLEDAI-2K, SLE Disease Activity Index.

### Comparisons between Q-SLAQ and SLAQ

An additional set of patients (n=85) completed both the Q-SLAQ and the SLAQ (table 5). Of these, 38 visits were with physicians who also assessed SLAM, and 47 were appointments without physicians, and consequently, SLAM was not performed. Bivariate correlation between Q-SLAQ and SLAQ were high both for the item patients global (ρ 0.96) and for the two summary scores (Symptom Score ρ 0.86 and total score ρ 0.82). In the subgroup of participants who also met a physician (n=38), the correlations between the SLAM-nolab and both the patient-reported Q-SLAQ and the SLAQ were generally good but in favour of the Q-SLAQ for both Symptom Score (ρ 0.70 vs ρ 0.56, p<0.001) and total score (ρ 0.77 vs ρ 0.64, p<0.001).

**Table 5:** Characteristics for the comparison between Q-SLAQ and SLAQ (n=85)

	Median	IQR	Min–Max
Age	45	33.0–53.5	19–78
Disease duration *	15	5–24	0–54
SLAM total †	6	3–10	1–17
SLAM-nolab †	4	2–8	0–13
SLAQ flare	1	0–1.5	0–3
SLAQ Patient’s Global Disease Activity	4	2–7	0–10
Q-SLAQ Patient’s Global Disease Activity	4	2–7	0–10
SLAQ Symptom Score	11	7.0–14.5	0–23
Q-SLAQ Symptom Score	9	6–12	0–18
SLAQ total score	13	7.5–19	0–31
Q-SLAQ total score	11	6–15	0–27

*Missing=14.

†Missing=47=patients without physicians’ assessment according to clinical practice.

Q-SLAQ, Quick Systemic Lupus Activity Questionnaire; SLAM, SLE Activity Measure; SLAM-nolab, Systemic Lupus Activity Measure without laboratory parameters; SLAQ, Systemic Lupus Activity Questionnaire.

## Discussion

In this study, we explored a revised and shorter version of the Swedish SLAQ, the Q-SLAQ. Despite an easier questionnaire with fewer evaluated items, we obtained numerically stronger correlations between physician’s SLAM and patients’ assessments of disease activity using Q-SLAQ than previously reported with the original SLAQ.^5 7 15^

The strongest correlation was obtained after omitting the tentative item sicca symptoms from the scoring of Q-SLAQ. Sicca symptoms were included in the preliminary version of the questionnaire after suggestions from patients and research partners. They emphasised the importance and distress of these symptoms and how they are often neglected by the healthcare.^16^ The majority of patients with SLE with sicca symptoms seem to belong to a subset with distinct clinical and laboratory features,^17^ often considered to have a milder version of SLE. We recently demonstrated that 23% of a large SLE cohort fulfils criteria for secondary Sjögren’s syndrome, and that this subgroup is affected by a pronounced systemic inflammation.^18^ Sicca symptoms are thus both common and disturbing for patients. But, as they are usually permanent, we think that they should be regarded as a manifestation of organ damage and not disease activity. Consequently, they should be collected as a separate item and not included in this type of summary score that aims to reflect patients’ assessment of disease activity.

We omitted the item stroke, which is included in SLAM, SLEDAI-2K and the original SLAQ.^5 8–10^ Strokes, together with other vascular events, for example, myocardial infarctions and deep venous thromboses, are over-represented in SLE.^19 20^ In our opinion, all these items reflect damage and should not be included in a disease activity instrument. Moreover, we think that it is inappropriate and may cause unnecessary anxiety to repeatedly ask patients if they have experienced a stroke in preparation for routine clinical visits or registry registrations.

A better item response rate in comparison to our previous SLAQ study in a similar context^7^ was another strength of Q-SLAQ. We believe that the present version of the questions may be easier to understand and answer than previous versions, giving the results stronger validity. Furthermore, the internal consistency results show that the strength of the Q-SLAQ is equal to or better than the previous versions.^7 15 21 22^ Additionally, the results of the correlation analyses were confirmed by comparing the results of the patients with low disease activity (SLAM score≤6) and those with high disease activity (SLAM score≥7), resulting in a distinct and significant difference between the two groups. Interestingly, the best correlations between patients and physicians were observed in the group with low disease activity. Conclusions from stratified analyses must, however, be interpreted with caution since the groups are small, for example, the high disease activity group consisted of 35 patients. However, one could speculate whether patients with less symptoms have a greater chance to discuss the actual symptoms during consultation times. High disease activity in SLE often involves several organs and more extensive need to examine them. Thus, with limited consultation time, there may not be enough time to discuss patients’ perceptions of all symptoms. Furthermore, patients with high disease activity may be occupied by a few dominating symptoms, while other symptoms may be neglected unless specifically asked for. Additionally, laboratory findings, for example, urinary casts, are silent to the patients and can therefore not be addressed by the patient. Further, the large contribution of laboratory result to the total SLEDAI-2K in the low disease group (SLAM score≤6) indicates the importance to add laboratory examinations even in patients with low disease activity. Discordance between patients’ and physicians’ assessment of disease activity has previously been identified.^23^ The good correlations between patients’ and physicians’ evaluations in the ‘low-active disease group’ support the possibility that telephone or digital consultations accompanied by Q-SLAQ and laboratory tests can replace some physical visits in selected patients. Though Q-SLAQ has several advantages over the SLAQ, especially in daily clinical practice, the original SLAQ may be more useful in epidemiological or multinational studies, particularly since the SLAQ is translated and validated to many languages.^5 7 15 21 22^

When comparing specific item correlations in the Q-SLAQ with corresponding item correlations in the Swedish SLAQ, seven items in this revised version correlated better between physicians’ and patients’ assessments.^7^ Interestingly, correlations between the specific organ items have been presented in some^15^ but not in all cultural validations of SLAQ.^21 22^ The results mirror discrepancies between two different perspectives and indicate gaps in the communication between patients and physicians, which in our study are most obvious regarding respiratory distress.^24 25^ Very weak to no correlation between the patients’ and the physicians’ assessments was found for dyspnoea/chest pain. Nevertheless, the Cronbach’s alpha did not suggest that the scale needed to be altered to improve internal consistency. In the original validation of SLAQ, patients also reported pulmonary distress frequently (50%), with low associations to the physicians’ assessments on this item.^5^ This discrepancy may be explained by the fact that the patients’ assessments reflect a broader set of common symptoms than the physicians’ assessments, which is confined to clinical definitions and signs of serositis. Notably, shortness of breath, also a symptom of heart disease, is not covered by either SLAM or SLEDAI-2K. According to our results, cardiopulmonary symptoms are relatively common in patients with SLE, and the low correlations found in the present study underscores the importance for clinicians to assess these symptoms more carefully, even early during the disease course.^26 27^ We believe that these findings are important and need further exploration.

SLE is a heterogeneous disease, and a complete understanding of its biological mechanisms is still lacking. Hence, evaluation of disease activity and severity of clinical manifestations is done by composite scores.^8^ Physicians’ assessments of disease activity in SLE and patients’ assessments of symptom distress are two perspectives that, when combined, reflect a more complete disease evaluation than if only one perspective is considered.^24^ Furthermore, collecting patients’ perspectives of symptoms facilitates interaction, reflection and ability to communicate symptoms and how they impact everyday life for patients.^28^ In the future, there is also a possibility to combine both perspectives with laboratory evaluations, where for example, measures of renal engagement are important, since they are not possible to capture by PROMs.

Further, our better correlations between Q-SLAQ and SLAM than with SLEDAI-2K demonstrate that the SLAM index includes a graded and broader range of SLE symptoms of importance to the patients, a previously discussed discrepancy.^29^ All these observations confirm the necessity to continue the discussion on how to differentiate disease activity from disease burden/impact and damage.^3^ These aspects are equally important but need different approaches and treatment actions.

We included 115 paired patient and physician assessments and we validated Q-SLAQ versus SLAQ in an additional set of 85 patients. A limited number, though our study comprises more participants than the first SLAQ study conducted by Karlson *et al*.^5^ The high response rate suggests that the questionnaire is easy to answer, which is a strength of this study. Test–retest analysis could not be performed to further validate the Q-SLAQ. This is recommended in future studies, and longitudinal studies are also needed to test how well Q-SLAQ can capture change in disease activity. Our additional data collection, with a clinical sample with and without physicians’ assessment, confirm that the Q-SLAQ present comparable data to SLAQ; however, we acknowledge that the sample is small.

We acknowledge that the original SLAQ,^5^ which is translated into several languages,^7 15 21 22^ is suitable for epidemiological studies with comparisons between countries. Nevertheless, the SLAQ is extensive, and in clinical everyday practice with frequent visits, we stress that a shorter questionnaire that covers a shorter period would be preferable. Additionally, we find that the care of today tends to include more and more digital contacts, and it is thus important to have validated patients’ assessments that we could use in a structured way. Since Q-SLAQ have the same structure and key elements as the SLAQ, we recommend Q-SLAQ rather than a completely different assessment.

To conclude, the performance of the shorter Q-SLAQ is similar to the original version of the SLAQ, demonstrating that it can be used to monitor disease activity in SLE. As we and others^5^ noted the substantial discrepancy between physicians’ and patients’ assessments of thoracic and respiratory pain/symptoms, further investigations of these items are clearly needed. We also explored the inclusion of sicca symptoms, based on patient suggestions, but believe that they are manifestations of damage rather than disease activity and should therefore not be part of Q-SLAQ. Overall, our results are encouraging and support the use of the Q-SLAQ in clinical care. We believe it is especially well suited in clinical situations when it is not possible to conduct physical examinations, for example, to support digital and telephone contacts, but also for registry purposes.

## Data Availability

Data are available upon reasonable request from the corresponding author: Elisabet Svenungsson, Karolinska University Hospital, Solna, S-171 76 Stockholm, Sweden (elisabet.svenungsson@ki.se).
