# Supplementary material for: Quick Systemic Lupus Activity Questionnaire (Q-SLAQ): a simplified version of SLAQ for patient-reported disease activity
Source: Lupus Sci Med. 2021 May 9;8(1):e000471. doi: 10.1136/lupus-2020-000471 (PMC8112425; doi:10.1136/lupus-2020-000471)
Supplement: Supplementary data [file lupus-2020-000471supp001.pdf]

Patients assessment of SLE activity using Q-SLAQ

Calculation of Quick-SLAQ (Q-SLAQ)

High values indicate greater perceived disease impact/distress.

The Total Q-SLAQ score is a summary score on a scale of 0–34

Q-SLAQ item (name of variable)	SLAQ response	Scoring Algorithm
Lost weight without trying	Mild<br>Moderate<br>Severe	1<br>2<br>3
Fatigue	Mild<br>Moderate<br>Severe	1<br>2<br>3
Fever (>38,5 ° C) taken by thermometer	Mild<br>Moderate<br>Severe	1<br>2<br>3
Swollen glands (nodes) in the neck (Lymphadenopathy)	Mild<br>Moderate<br>Severe	1<br>2<br>3
Skin/mucosal<br>-Rash on cheeks (shape like a butterfly)<br>-Rash or feeling sick after going out in the sun<br>-Sores in mouth or nose	Score any positive response as one item with max score of 1	1
Alopecia<br>(Bald patches on scalp, or clumps of hair on pillow)	Mild<br>Moderate<br>Severe	1<br>2<br>3
Pulmonary distress:<br>-Shortness of breath<br>-Chest pain w/deep breath	Score as one item using highest score on either item (pulmonary)	1<br>2<br>3
Abdominal pain	Mild<br>Moderate<br>Severe	1<br>2<br>3
Cognitive distress:<br>-Forgetfulness<br>-Feeling depressed	Score as one item using highest score on either item (cognitive impairment)	1<br>2<br>3
Unusual headaches	Mild<br>Moderate<br>Severe	1<br>2<br>3
Muscle weakness (A)<br>Muscle pain (B)	Score A as primary, mild/moderate/severe<br>If A=0, score B as mild/moderate/severe	1/2/3
Joint pain or stiffness (B)<br>Joint swelling (A)	Score A as primary, mild/moderate/severe<br>If A=0, score B as mild/moderate/severe	1/2/3

Svenungsson E, *et al. Lupus Sci Med* 2022; 8:e000471. doi: 10.1136/lupus-2020-000471

Patients assessment of SLE activity using Q-SLAQ

The Symptom score is a summary score resulting in a possible score of 0-18

**SLAQ item (name of variable)**	**Any positive response (mild/moderate/severe)**	**No positive response**
Weight loss, unintentional	1	0
Fatigue	1	0
Fever (>38,5 ° C)	1	0
Lymphadenopathy	1	0
Rash on cheeks (shape like a butterfly	1	0
Rash or feeling sick after going out in the sun	1	0
Sores in mouth or nose	1	0
Alopecia	1	0
Shortness of breath	1	0
Chest pain w/deep breath	1	0
Abdominal pain	1	0
Forgetfulness	1	0
Signs of depression	1	0
Unusual headaches	1	0
Muscle weakness	1	0
Muscle pain	1	0
Join pain or stiffness	1	0
Swollen joints	1	0

The Patient's global disease activity

Patients assessment of SLE disease activity during the last month scored on a numeric rating scale from 0 (no activity) to10 (most activity).

Svenungsson E, *et al. Lupus Sci Med* 2022; 8:e000471. doi: 10.1136/lupus-2020-000471
