# Supplementary material for: Quick Systemic Lupus Activity Questionnaire (Q-SLAQ): a simplified version of SLAQ for patient-reported disease activity
Source: Lupus Sci Med. 2021 May 9;8(1):e000471. doi: 10.1136/lupus-2020-000471 (PMC8112425; doi:10.1136/lupus-2020-000471)
Supplement: Supplementary data [file lupus-2020-000471supp002.pdf]

Supplement Results from Q-SLAQ, SWE-SLAQ [1] and SLAQ [2 3]

	Q-SLAQ n=115	SWE-SLAQ [1] n=203	SLAQ [2] n=93
Cronbach alpha			
Total Q-SLAQ	0.89	0.86	0.87 [3]
Symptom score	0.91	0.91	-
Correlation, SLAM-nolab versus:			
Total Q-SLAQ	0.71	0.69	0.62 [2]
Symptom score	0.68	0.65	0.54 [2]
Patient global (NRS)	0.68	0.60	0.52 [2]

Abbreviations:

NRS= numeric rating scale

SLAM-nolab = Systemic Lupus Activity Measure without laboratory parameters

SLAQ = Quick Systemic Lupus Activity Questionnaire

SWE-SLAQ = Swedish version of SLAQ

Q-SLAQ = Quick Systemic Lupus Activity Questionnaire (shorter version of SLAQ)

References:

1. Pettersson S, Svenungsson E, Gustafsson J, et al. A comparison of patients' and physicians' assessments of disease activity using the Swedish version of the Systemic Lupus Activity Questionnaire. *Scand J Rheumatol* 2017;46(6):474-83.
2. Karlson EW, Daltroy LH, Rivest C, et al. Validation of a Systemic Lupus Activity Questionnaire (SLAQ) for population studies. *Lupus* 2003;12(4):280-6.
3. Yazdany J, Yelin EH, Panopalis P, et al. Validation of the systemic lupus erythematosus activity questionnaire in a large observational cohort. *Arthritis Rheum* 2008;59(1):136-43.
